# Overexpression of *rttA* leads to activation of the fumicycline biosynthesis cluster including the O-acetyltransferase encoding *fccF*

**DOI:** 10.1128/spectrum.02288-26

**Published:** 2026-09-04

**Authors:** Lukas Birštonas, Sophie Tröger-Görler, Ingo Bauer, Birte Mertens, Henrick Schweder, Klaus Faserl, Axel A. Brakhage, Fabio Gsaller

**Affiliations:** 1 Institute of Molecular Biology, Biocenter, Medical University of Innsbruck 27280, Innsbruck, Austria; 2 Department of Molecular and Applied Microbiology, Leibniz Institute for Natural Product Research and Infection Biology (Leibniz-HKI) 28406, Jena, Germany; 3 Institute of Microbiology, Friedrich Schiller University (FSU) 163335, Jena, Germany; 4 Protein Core Facility, Institute of Medical Biochemistry, Biocenter, Medical University of Innsbruck 27280, Innsbruck, Austria; Brown University, Providence, Rhode Island, USA

**Keywords:** *Aspergillus fumigatus*, transcriptional regulation, secondary metabolites, fumicycline (neosartoricin) biosynthetic cluster, O-acetyltransferase

## Abstract

The opportunistic mold *Aspergillus fumigatus*, a WHO-priority fungal pathogen, causes life-threatening invasive infections in immunocompromised patients. A major antifungal drug target is the ergosterol biosynthesis pathway, primarily regulated by the transcription factors SrbA and AtrR. Recently, we identified RttA as an additional regulator of sterol homeostasis that mediates the activation of the sterol C24-methyltransferase-encoding gene *erg6*. Here, we demonstrate that overexpression of *rttA* leads to elevated production of the secondary metabolite fumicycline via induction of *fccR*, coding for the principal transcriptional activator of fumicycline biosynthesis. Lack of *fccR* blocked activation of fumicycline biosynthetic genes (*fccA-fccE*) during *rttA* overexpression, highlighting the indispensable role of FccR for activation of this cluster. In contrast, *rttA* inactivation did not abolish fumicycline production during *fccR* overexpression or during co-cultivation with *Streptomyces rapamycinicus*. In addition to *fccA-fccE*, our findings reveal a crucial role of FccR for the upregulation of a gene located adjacent to the fumicycline biosynthesis cluster, here termed *fccF*. Deletion of *fccF* severely diminished the conversion of fumicycline B into fumicycline C, uncovering FccF as fumicycline B O-acetyltransferase. Intriguingly, during *fccR* overexpression and resulting fumicycline overproduction, inactivation of *fccF* caused growth defects, likely due to precursor toxicity. Together, these findings broaden our understanding of fungal secondary metabolite regulation and reveal a novel role of RttA in the activation of *fccR* encoding the cluster-specific regulator that activates fumicycline production, including FccF, the terminal acetyltransferase in the pathway.

**IMPORTANCE:**

*Aspergillus fumigatus* is a major opportunistic fungal pathogen that causes severe infections in immunocompromised individuals and represents a significant global health threat. Understanding the regulatory networks controlling fungal metabolism is important for identifying potential therapeutic vulnerabilities. Here, we uncover a previously unrecognized role for the sterol homeostasis regulator RttA in activating fumicycline production through induction of the pathway-specific transcription factor FccR. We further show that FccR controls expression of an additional biosynthetic gene, *fccF*, and identify FccF as the terminal O-acetyltransferase required for conversion of fumicycline B to fumicycline C. Disruption of this final biosynthetic step impairs fungal growth during metabolite overproduction, indicating that accumulation of fumicycline B or related intermediates can negatively affect fungal growth. Together, these findings expand our understanding of fungal secondary metabolite regulation and provide new insights into the organization and function of the fumicycline biosynthetic pathway.

## OBSERVATION

Recent estimates indicate that fungal infections are linked to more than 3.8 million human deaths annually, far exceeding previous estimates of 1.5–2.0 million cases of fatality per year (1–3). Aspergillosis, a severe disease caused by species of the genus *Aspergillus*, accounts for more than 2 million of these deaths (3). Among *Aspergillus* species, the human pathogenic mold *Aspergillus fumigatus* is the primary causative agent of aspergillosis and has been designated by the World Health Organization (WHO) as one of the four critical priority fungal pathogens (4).

**Fig 1:**
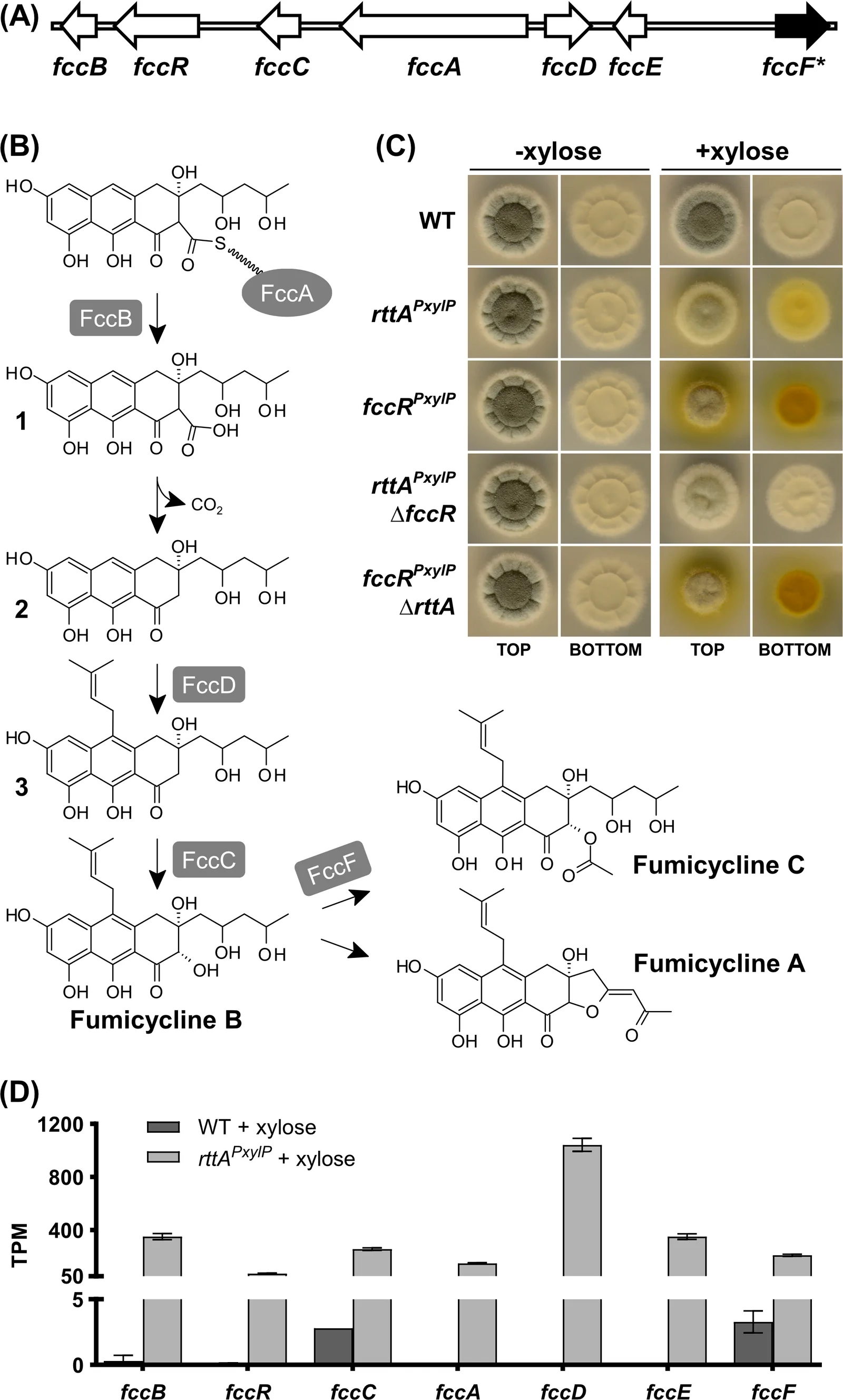
Induction of *rttA* activates expression of the fumicycline secondary metabolite gene cluster (SMC)**.** (**A**) Schematic of the fumicycline SMC. (**B**) Biosynthesis of fumicycline A, fumicycline B, and fumicycline C. (**C**) Phenotypic analysis of *PxylP*-inducible *rttA* and *fccR* mutants during non-inducing (−xylose, 0% xylose) and inducing (+xylose, 1% xylose) conditions. Strains were grown on solid AMM for 48 h at 37°C. For each condition, the first column shows colonies imaged from above (TOP), while the second column shows the corresponding colonies imaged from below (BOTTOM). (**D**) RNAseq-based expression analysis of fumicycline biosynthetic genes during *rttA* overexpression. For expression analysis, strains were grown in AMM +1% xylose for 18 h at 37°C. The data represent the relative transcript abundance (TPM, transcript per million) derived from DESeq2 analysis of biological triplicates. *The respective gene has not been characterized prior this work.

Detrimental effects of aspergillosis result from a combination of physical invasion and disruption of the host’s immune system and homeostasis (5). *A. fumigatus* has the capacity to produce an array of secondary metabolites such as siderophores, gliotoxin, and DHN-melanin that contribute to the success of infection (6–8). Genes associated with such metabolic pathways are frequently co-localized within so-called secondary metabolite gene clusters (SMCs), which are typically located in transcriptionally silent genomic regions (9, 10). Some fungal SMCs are known to be activated in response to specific environmental stresses or upon exposure to particular molecules or surrounding bacteria (11–13). One of the SMCs shown to be induced upon co-cultivation of *A. fumigatus* with the bacterial species *Streptomyces rapamycinicus* is the fumicycline (neosartoricin) biosynthetic cluster (Fig. 1A), which was first discovered in *Neosartorya fischeri* and *A. fumigatus* during genome mining for polycyclic prenyltransferases (14, 15). The induced compounds exhibited only moderate activity against this bacterial species (15). In addition to co-cultivation with bacteria, upregulation of this cluster was observed in *A. fumigatus* during murine lung infection and in an *fphB* phytochrome gene deletion mutant (16, 17). In recent work, the arginoketide azalomycin F, a bacterial metabolite produced and excreted by *Streptomyces iranensis*, was shown to activate the cluster (12). In previous work, antiproliferative activity *in vitro* against anti-CD3/CD28-activated murine spleenic T cells was detected, implying an immunosuppressive role of fumicycline C (14). A clear inhibitory potential of fumicycline C against Gram-positive and -negative bacteria, yeast species *Saccharomyces cerevisiae* and *Candida albicans,* A549 lung epithelial cells, and RAW 264.7 macrophage cells could not be identified (14).

**Fig 2:**
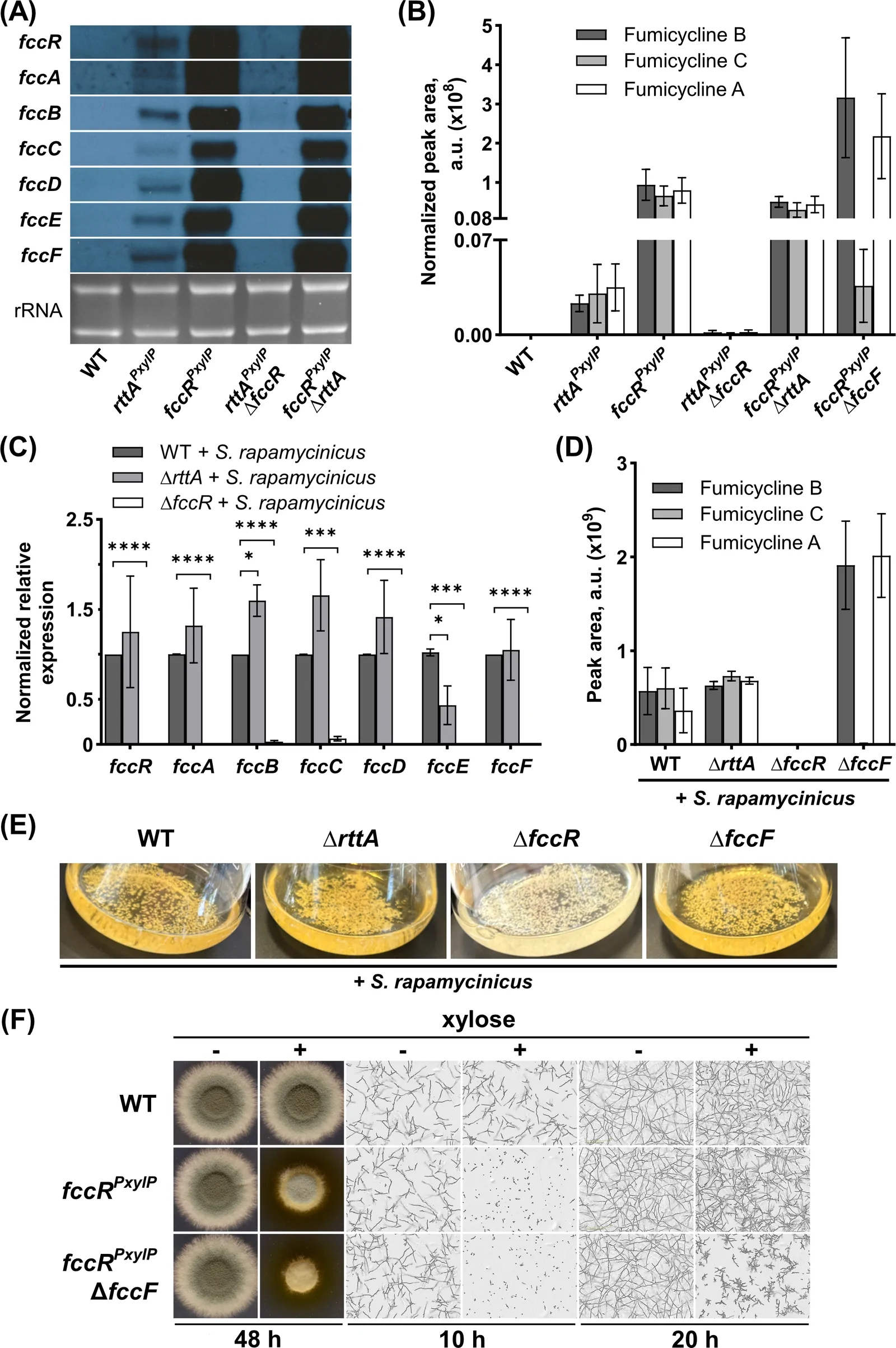
Induction of the fumicycline SMC through *rttA* overexpression or co-cultivation with *S. rapamycinicus* relies on FccR. (**A**) Northern blot-based expression analysis of fumicycline biosynthetic genes. rRNA visualized by ethidium bromide staining is shown to assess RNA quality and loading consistency. Strains were grown in liquid AMM +1% xylose for 18 h at 37°C, 200 rpm. (**B**) LC-MS-based quantification of fumicycline abundance in mutant strains during *rttA* and *fccR* overexpression. Strains were grown in liquid AMM +1% xylose for 24 h at 37°C, 200 rpm. Values normalized to corresponding biomass generated during growth. (**C**) RT-qPCR-based expression analysis of the fumicycline SMC genes in WT, ∆*rttA*, and ∆*fccR* in *S. rapamycinicus* co-culture. The expression levels of target genes were compared with the expression of these genes in the WT and normalized on the expression of *act*1 using the 2^−∆∆Ct^ method. Welch's *t*-test was used to test significance. Significances were included as follows: *P < 0.05, ***P < 0.001, ****P < 0.0001. (**D**) LC-MS-based detection of fumicycline levels in WT and deletion strains in *S. rapamycinicus* co-culture. Fungal pre-cultures were grown in liquid AMM for 16 h at 37°C, 200 rpm. Main cultures were co-cultured in liquid AMM with *S. rapamycinicus* for 8 h at 37°C, 200 rpm. (**E**) Images displaying fungal cultures of mutant strains after co-cultivation with *S. rapamycinicus.* (**F**) Phenotypic and microscopic analysis comparing growth of *fccR^PxylP^* with *fccR^PxylP^*∆*fccF* during non-inducing (−xylose, 0% xylose) and inducing (+xylose, 1% xylose) conditions. For radial growth assays, strains were grown on solid AMM for 48 h at 37°C. For microscopic analysis, strains were grown in liquid AMM for 20 h at 37°C. Micrographs were acquired after 10 and 20 h.

Fumicycline biosynthesis involves the consecutive action of the enzymes FccA-FccD (Fig. 1B), which leads to the generation of fumicycline B. In a final step of the pathway, fumicycline B undergoes acetylation to form fumicycline C. Alternatively, dehydration of fumicycline B through the loss of one water molecule leads to the formation of fumicycline A (14, 15). FccR represents the principal transcriptional activator of the fumicycline biosynthetic cluster, and its encoding gene is located within the cluster itself. It has been demonstrated that its overexpression increases the expression of all fumicycline biosynthetic genes within the cluster (14, 15). In this study, we demonstrate that genes of the fumicycline biosynthetic cluster can also be activated by overexpressing *rttA*, a previously characterized transcription factor involved in the maintenance of sterol homeostasis and azoleresistance (18, 19). Moreover, we identify a previously uncharacterized gene adjacent to the fumicycline biosynthetic gene cluster and demonstrate its role in fumicycline biosynthesis. During overexpression of *rttA*, we observed unusual yellow pigmentation in both the mycelium and the surrounding medium (Fig. 1C). Hence, we suspected upregulation of a biosynthetic pathway in this mutant responsible for overproduction of a specific metabolite. To elucidate corresponding differentially expressed genes linked to a biosynthetic cluster, we performed RNAseq analysis comparing transcriptional profiles of wild type (WT) and *rttA^PxylP^* (mutant carrying an additional xylose-inducible *rttA* copy) grown in the presence of 1% xylose. Among the genes that showed the highest differential expression in *rttA^PxylP^* versus WT, we detected all the genes of the fumicycline SMC (Fig. 1D). Therefore, we suspected that the observed yellow pigmentation results from overproduction of fumicyclines, which would be in agreement with previous work reporting this phenotype (14). To investigate whether *rttA* overexpression could be responsible for upregulation of the individual biosynthetic genes inside the cluster directly, independent of *fccR*, we deleted *fccR* in *rttA^PxylP^*. The resulting strain *rttA^PxylP^*Δ*fccR* exhibited no yellow pigmentation during inducing conditions (Fig. 1C), indicating that overexpression of *rttA* leads to induction of *fccR*, which in turn activates the fumicycline SMC. To exclude a direct contribution of RttA to fumicycline production during *fccR* overexpression, we generated the reciprocal mutant *fccR^PxylP^*Δ*rttA*. There was no observable difference in the yellow pigmentation comparing *fccR^PxylP^*Δ*rttA* with *fccR^PxylP^* (Fig. 1C). In addition to the phenotypic analysis, we monitored the expression of the fumicycline SMC genes using northern blot analysis (Fig. 2A). During inducing conditions, the fumicycline biosynthetic genes *fccA-fccE* were upregulated in *rttA^PxylP^*, *fccR^PxylP^*, and *fccR^PxylP^*Δ*rttA* but not *rttA^PxylP^*Δ*fccR*. Notably, compared with *rttA*, upregulation of *fccR* led to a substantially higher expression of the genes. Using LC-MS analysis, we detected a high abundance of fumicyclines in the strains with elevated transcript levels of *fccA-fccE* (Fig. 2B). The lack of *fccA-fccE* expression and abolished metabolite production in *rttA^PxylP^*Δ*fccR* further confirms the indispensable role of FccR for activation of the fumicycline SMC. In addition to xylose-induced artificial activation of the cluster through *rttA* and *fccR* overexpression, we generated single deletion mutants Δ*rttA* and Δ*fccR* to investigate the contribution of these genes to cluster activation during co-cultivation with *S. rapamycinicus*. Consistent with a dispensable role of RttA, neither expression of fumicycline cluster genes nor production of fumicyclines was diminished during co-cultivation with ∆*rttA* compared with WT (Fig. 2C through E). To date, the mechanism underlying *fccR* induction during *rttA* overexpression remains to be identified. Although speculative, *rttA* overexpression may perturb sterol homeostasis and thereby alter membrane composition or membrane-associated stress signaling, indirectly elevating *fccR*, which results in the activation of fumicycline biosynthetic genes. Consistent with a membrane-stress linked route to *fccR* activation, amphotericin B, which targets the fungal membrane by binding ergosterol and forming pores, induces expression of *eneA*, encoding a transcription factor involved in the expression of fumicycline biosynthetic genes (20). Future work will test whether RttA directly activates *fccR* via promoter binding or regulates it indirectly, and will aim to elucidate the triggers that lead to *fccR* activation via RttA.

In addition to *fccA-fccE*, a gene located directly downstream of the fumicycline SMC (AFUB_086740) (Fig. 1A), here designated *fccF*, was strongly upregulated in *rttA^PxylP^* and *fccR^PxylP^* during inducing conditions (Fig. 1D and Fig. 2A through C). Using the translated FccF protein sequence as input for the InterPro (21) classification tool, FccF was annotated as a putative “plant and fungal acyltransferase.” Domain architecture analysis predicted a chloramphenicol acetyltransferase-like domain, while family classification assigned the protein to the trichothecene 3-O-acetyltransferase family (IPR050317). Based on this, we hypothesized that FccF is responsible for the acetylation of fumicycline B to fumicycline C. Prior to this study, the gene and corresponding enzyme catalyzing this reaction had not been identified. To investigate the potential involvement of FccF in this reaction, we deleted the gene encoding FccF in the *fccR^PxylP^* background (strain *fccR^PxylP^*Δ*fccF*) and detected fumicycline levels in the supernatants of the mutant strains using LC-MS (Fig. 2B). Comparison of *fccR^PxylP^* with *fccR^PxylP^*Δ*fccF* showed that production of fumicycline C was almost abolished, accompanied by increased levels of fumicycline B. This is consistent with the predicted role of FccF as the O-acetyltransferase catalyzing the conversion of fumicycline B to fumicycline C. Moreover, fumicycline A levels were elevated in the absence of FccF, which may result from increased availability of substrate for dehydration. Similar results were observed during co-cultivation of ∆*fccF* with *S. rapamycinicus* (Fig. 2D). These findings confirm the role of FccF as O-acetyltransferase responsible for acetylation of fumicycline B. Interestingly, radial growth as well as microtiter-based microscopic analysis revealed a growth impairment of *fccR^PxylP^*Δ*fccF* compared with *fccR^PxylP^* (Fig. 2F). Notably, microscopic analysis showed more pronounced growth differences between the mutants at later time points (10 h vs 20 h), which may result from accumulation of fumicycline B and/or fumicycline A over time (Fig. 2F).

Together, these data demonstrate that overexpression of *rttA* upregulates *fccR*, which in turn activates the fumicycline SMC, including *fccF*, here characterized as encoding the O-acetyltransferase catalyzing the conversion of fumicycline B to fumicycline C. Disruption of this step exacerbates growth defects associated with fumicycline production, likely due to markedly reduced fumicycline C levels and consequent accumulation of its precursor fumicycline B and/or fumicycline A.

Detailed information on the Materials and Methods is provided in the Supplemental material accompanying this manuscript.

## Data Availability

All next-generation sequencing data are available through the BioProject PRJNA1481417.

## SUPPLEMENTAL MATERIAL

Supplemental materials spectrum.02288-26-s0001.pdf Additional experimental details, Table S1 and S2, and Fig. S1 and S2.

ASM does not own the copyrights to Supplemental Material that may be linked to, or accessed through, an article. The authors have granted ASM a non-exclusive, world-wide license to publish the Supplemental Material files. Please contact the corresponding author directly for reuse.
