# Supplementary material for: Overexpression of rttA leads to activation of the fumicycline biosynthesis cluster including the O-acetyltransferase encoding fccF
Source: Microbiol Spectr. 2026 Sep 4;14(10):e02288-26. doi: 10.1128/spectrum.02288-26 (PMC13688909; doi:10.1128/spectrum.02288-26)
Supplement: Supplementary file 1 [file spectrum.02288-26-s0001.pdf]

Supplemental Information

Materials and Methods

Generation of strains

The strains used in this study are listed in Table S1, and the primers used are listed in Table S2. To construct *rttA* and *fccR* overexpression strains, a *PxylP*-containing linear backbone was PCR-amplified from pΔazgA-Luc_{Opt}^{PxylP} (1) using primers pX-FW.2/RV.2. The genomic regions of *rttA* and *fccR* were amplified from genomic DNA with primer pairs rttA-FW/RV and fccR-FW/RV, respectively. Inserts bearing overlaps to the backbone were assembled into the backbone using the NEBuilder HiFi DNA Assembly Master Mix (New England Biolabs Inc., Ipswich, MA, USA). The resulting plasmids, pΔazgA-$rttA^{PxylP}$ and pΔazgA-$fccR^{PxylP}$ (Fig. S1), were linearized with *Not*I and used to transform *A. fumigatus* A1160P+. The *Not*I-linearized pΔazgA-$fccR^{PxylP}$ plasmid was additionally used to transform the Δ*rttA* strain (2).

To generate the $rttA^{PxylP}\Delta fccR$ and Δ*fccR* knockouts, the *fccR* coding region in the $rttA^{PxylP}$ and WT backgrounds was replaced with a hygromycin B resistance cassette. Approximately 1 kb fragments flanking the 5′ and 3′ ends of *fccR* were PCR-amplified from *A. fumigatus* genomic DNA using primer pairs fccR-1/2 and fccR-3/4. Using primers hph-FW/RV, we amplified the hygromycin B resistance cassette from the pAN7-1 plasmid (3). The two flanks were then joined to the resistance cassette by fusion PCR, as previously described (4) using nested primers fccR-N1/N2 to generate the deletion construct. $rttA^{PxylP}$ and *A. fumigatus* A1160P+ were subsequently transformed with the prepared deletion cassette (Fig. S1).

To construct the $fccR^{PxylP}\Delta fccF$ and Δ*fccF* knockouts, the *fccF* coding region in the $fccR^{PxylP}$ and WT backgrounds was replaced with a hygromycin B resistance cassette. Approximately 1 kb fragments flanking the 5′ and 3′ ends of *fccF* were PCR-amplified from *A. fumigatus* genomic DNA using primer pairs fccF-1/2 and fccF-3/4. The hygromycin B resistance cassette was amplified as described above. For the fusion PCR, joining the resistance cassette to the flanks, nested primers fccF-N1/N2 were employed. $fccR^{PxylP}$ and *A. fumigatus* A1160P+ were subsequently transformed with the *fccF* deletion cassette (Fig. S1).

Following previously described methods (1, 4) we performed fungal transformations under 8-azaguanine or hygromycin B selection and confirmed all strains by PCR (Fig. S2).

Metabolite analysis with liquid-chromatography coupled to mass-spectrometry (nanoLC-MS/MS).

100 mL of *Aspergillus* liquid minimal medium (AMM) (5) in conical 500 mL flasks was used. Each flask was inoculated with 1×10^8 spores and then incubated at 37 °C in an orbital shaker at 200 rpm. As inducer (for *rttA*^{PxylP} and *fccR*^{PxylP}), xylose was added to all flasks to a final concentration of 1% (wt/vol) prior to incubation. After 24 hours incubation, supernatant and biomass were collected by filtration as separate fractions. Biomass was freeze-dried and weighed. 4 mL of the supernatant was used for metabolite extraction with 1 mL of ethyl acetate (EtOAc)/methanol (MeOH)/acetic acid (AcOH) (89:10:1). The mixture was vortexed for 10 seconds and centrifuged for 5 minutes at 3000 rpm. 100 µL of organic phase was then transferred to a fresh tube. Samples were diluted 1:10 with H_2O and 2 µL were used for analysis. Extracts were analyzed using an UltiMate 3000 nano-HPLC system coupled to a Q Exactive Plus mass spectrometer (Thermo Scientific). Analytes were separated on a 17 cm long column (100 µm i.d.) packed with 2.4 µm C18 material (Reprosil). Separation was performed using a linear gradient of 4–85% acetonitrile (CH_3CN) in water (0.1% formic acid) for 30 min followed by an isocratic step at 85% CH_3CN in water (0.1% formic acid) for another 15 min. Flow rate was set to 0.3 mL/min. Full MS scans were acquired from 300 to 500 *m/z* at a resolution of 60,000. The 20 most abundant precursor ions from the full MS scan were selected for HCD fragmentation using an isolation window of 1.6 m/z and a normalized collision energy of 28, with MS/MS spectra acquired at a resolution of 30,000.

All experiments were performed in triplicate.

LC-MS based quantifications in co-culture

Co-cultivation of *Streptomyces rapamycinicus* with *A. fumigatus* was conducted as described before (6). Pre-cultures of mutant strains and WT were grown for 16 h in liquid AMM at 37 °C, 200 rpm. Co-cultures of fungal strains and bacterium were photographed and harvested after 8 h of co-culture and

subsequently extracted for LC-MS analysis. Extraction and detection of secondary metabolites was carried out as described before (7, 8).

Radial growth assays and microscopical analysis

For the assessment of radial expansion, conidia were prepared in a water saline-detergent solution (0.9% NaCl, 0.1% Tween 20) and then 5 µL (containing 1×10^4 spores) were used for point-inoculation. The strains were cultured on AMM solidified with 1.5% (wt/vol) agar and enriched with 10 mM ammonium tartrate dibasic and 1% (wt/vol) glucose. For assays with Δ*fccF*, the pH of the medium was stabilized by supplementing the medium with 0.1 M of MOPS pH7. After 48 hours of growth at 37 °C, the resulting colonies were photographed for analysis.

For microscopy-based phenotypic analysis, same AMM liquid media with 1M MOPS (pH 7) buffer was used. Culturing was performed in microtiter 96-well format plates, each well containing 100 µl of media with 1×10^4 spores. For induction 1% (wt/vol) xylose was used. Images were taken after 10 and 20 hours of incubation at 37 °C.

Gene expression analysis

For northern blot analysis, *A. fumigatus* strains were inoculated at 1×10^6 spores/mL in 100 mL of AMM in a 500 mL conical flask and then incubated at 37 °C in an orbital shaker at 200 rpm. As inducer (for $rttA^{PxylP}$ and $fccR^{PxylP}$), xylose (1% final concentration, wt/vol) was added to all the flasks prior to incubation. After 18 hours incubation, biomass was separated from supernatant by filtration, shock-frozen and freeze-dried. 10 mg of pulverized biomass was used for total RNA extraction using TRI Reagent (Sigma-Aldrich, St. Louis, Missouri, USA) according to the manufacturer's recommendations. Northern blot analysis was performed as previously described (9).

For RT-qPCR of the fumicycline SMC, *A. fumigatus* strains were grown as pre-cultures in liquid AMM for 16 h at 37 °C, 200 rpm. Main cultures were co-cultivated with *S. rapamycinicus* as described before (6). The cultures were harvested after 3 h of co-cultivation. RNA isolation, cDNA synthesis and RT-qPCR were conducted as described before (10). As a reference gene for normalization, *act1* was used,

employing the 2^-ΔΔCt method (11). The sequences of RT-qPCR primers for *fccF* (fccF-RT-FW/RV) are listed in Table S2, primer sequences for other target genes can be found in (8).

RNAseq analysis

Following enrichment of Poly-(A)-tailed mRNA, directional libraries were sequenced on a NovaSeq X Plus instrument (Novogene GmbH) using a 150 bp paired-end strategy. Raw sequencing data was processed through the nf-core/rnaseq workflow (v3.17.0) (12), which utilized STAR (v2.7.11b) for alignment against the *Aspergillus fumigatus* A1163 genome (FungiDB, release 68) (13) and Salmon (v1.10.3) for quantification within a Singularity environment (14). Differential expression analysis was performed with DESeq2 (v1.49.0) in R (v4.5.0), while filtering relied on dplyr (v1.1.4alignments in IGV (v2.16.0).

References

1. Sastré-Velásquez LE, Mach N, Mertens B, Kühbacher A, Merschak P, Dallemulle A, Lechner L, Baldin C, Diallinas G, Gsaller F. 2025. Simultaneous multigene integration in *Aspergillus fumigatus* using CRISPR/Cas9 and endogenous counter-selectable markers. J Biol Eng 19:69.
2. Birštonas L, Hortschansky P, Bauer I, Alcanzo EM, Kühbacher A, Mertens B, Müller C, Brakhage AA, Gsaller F. 2025. The transcription factor RttA contributes to sterol regulation and azole resistance in *Aspergillus fumigatus*. mBio 16:e0185425.
3. Kubodera T, Yamashita N, Nishimura A. 2002. Transformation of *Aspergillus* sp. and *Trichoderma reesei* using the pyrithiamine resistance gene (*ptrA*) of *Aspergillus oryzae*. Biosci Biotechnol Biochem 66:404-6.
4. Fraczek MG, Bromley M, Buied A, Moore CB, Rajendran R, Rautemaa R, Ramage G, Denning DW, Bowyer P. 2013. The *cdr1B* efflux transporter is associated with non-*cyp51a*-mediated itraconazole resistance in *Aspergillus fumigatus*. J Antimicrob Chemother 68:1486-96.
5. Pontecorvo G, Roper JA, Hemmons LM, Macdonald KD, Bufton AW. 1953. The genetics of *Aspergillus nidulans*. Adv Genet 5:141-238.
6. Schroeckh V, Scherlach K, Nützmann HW, Shelest E, Schmidt-Heck W, Schuemann J, Martin K, Hertweck C, Brakhage AA. 2009. Intimate bacterial-fungal interaction triggers biosynthesis of archetypal polyketides in *Aspergillus nidulans*. Proc Natl Acad Sci U S A 106:14558-63.
7. Stroe MC, Netzker T, Scherlach K, Krüger T, Hertweck C, Valiante V, Brakhage AA. 2020. Targeted induction of a silent fungal gene cluster encoding the bacteria-specific germination inhibitor fumigermin. eLife 9:e52541.
8. Tröger-Görler S, Vij R, Schäuble S, Rosin M, Schweder H, Hortschansky P, Schroeckh V, Barber AE, Kniemeyer O, Brunke S, Panagiotou G, Hube B, Brakhage AA. 2026. The fungal transcription factor SmpR coordinates secondary metabolism and antibacterial defence in *Aspergillus fumigatus* during interspecies interaction. bioRxiv doi:10.64898/2026.04.05.716574.
9. Hortschansky P, Eisendle M, Al-Abdallah Q, Schmidt AD, Bergmann S, Thön M, Kniemeyer O, Abt B, Seeber B, Werner ER, Kato M, Brakhage AA, Haas H. 2007. Interaction of HapX with the CCAAT-binding complex--a novel mechanism of gene regulation by iron. Embo j 26:3157-68.
10. Abou-Kandil A, Tröger-Görler S, Pschibul A, Krüger T, Rosin M, Schmidt F, Akbarimoghaddam P, Sarkar A, Cseresnyés Z, Shadkchan Y, Heinekamp T, Gräler MH, Barber AE, Walther G, Figge MT, Brakhage AA, Osherov N, Kniemeyer O. 2025. The proteomic response of *Aspergillus*

fumigatus to amphotericin B (AmB) reveals the involvement of the RTA-like protein RtaA in AmB resistance. Microlife 6:uqae024.
11. Livak KJ, Schmittgen TD. 2001. Analysis of relative gene expression data using real-time quantitative PCR and the 2(-Delta Delta C(T)) Method. Methods 25:402-8.
12. Ewels PA, Peltzer A, Fillinger S, Patel H, Alneberg J, Wilm A, Garcia MU, Di Tommaso P, Nahnsen S. 2020. The nf-core framework for community-curated bioinformatics pipelines. Nat Biotechnol 38:276-8.
13. Basenko EY, Shanmugasundram A, Böhme U, Starns D, Wilkinson PA, Davison HR, Crouch K, Maslen G, Harb OS, Amos B, McDowell MA, Kissinger JC, Roos DS, Jones A. 2024. What is new in FungiDB: a web-based bioinformatics platform for omics-scale data analysis for fungal and oomycete species. Genetics 227.
14. Kurtzer GM, Sochat V, Bauer MW. 2017. Singularity: Scientific containers for mobility of compute. PLoS One 12:e0177459.

Supplemental Tables and Figures

Table S1. **Strains used in this study.** A1160P+ served as the recipient for all mutants generated and used in this study.

**Strain**	**Genotype**	**Reference**
A1160P+ (wt)	*Δku80::pyrG*	(4)
$rttA^{PxylP}$	*ΔazgA::PxylP-rttA*	This study
$fccR^{PxylP}$	*ΔazgA::PxylP-fccR*	This study
$rttA^{PxylP}\Delta fccR$	*ΔazgA::PxylP-rttAΔfccR::hph*	This study
$fccR^{PxylP}\Delta rttA$	*ΔazgA::PxylP-fccRΔrttA::hph*	This study
$fccR^{PxylP}\Delta fccF$	*ΔazgA::PxylP-fccRΔfccF::hph*	This study
ΔrttA	*ΔrttA::hph*	(2)
ΔfccR	*ΔfccR::hph*	This study
ΔfccF	*ΔfccF::hph*	This study

Table S2. **Primers used in this study.**

**Primer name**	**Sequence (5′ → 3′)**
pX-FW.2	CCATGGCAGCAGTGATTTCA
pX-RV.2	GGTTGGTTCTTCGAGTCGATG
rttA-FW	ATCGACTCGAAGAACCAACCATGCCACCGCGTCGCAGT
rttA-RV	TGAAATCACTGCTGCCATGGCTAGCTGTTGAGATCGACTGCCT
fccR-FW	ATCGACTCGAAGAACCAACCATGGAGAAAAGTAATCGGCG
fccR-RV	TGAAATCACTGCTGCCATGGCTAAGGAAGAAGCTGGTTCG
fccR-1	CTGTCCCACAAAGCATACAGTAGT
fccR-2	TAGTTCTGTTACCGAGCCGGTGGACTGCGTCTCTCCGT
fccR-3	GCTCTGAACGATATGCTCCCTGCTTTAACTGACCAGACAAGCG
fccR-4	ATCTATCCCGACGAGAAATAGACAAGA
hph-FW	CCGGCTCGGTAACAGAACTAACGGCGTAACCAAAAGTCAC
hph-RV	GGGAGCATATCGTTCAGAGCTCTTGACGACCGTTGATCTG
fccR-N1	GGTTGGGTGGCTGGCAGA
fccR-N2	CGTCCTTGAGCATAGATAAGGTTACGG
fccF-1	GCATGGCTGATAATGATTCTTGCAGA
fccF-2	TAGTTCTGTTACCGAGCCGGTGATCGGTGTAGGTGGCAGATAG
fccF-3	GCTCTGAACGATATGCTCCCCAAGCCTTCGAGCTGAGAAGTG
fccF-4	GCTCAACTCGAAGCAATCTGAGAT
fccF-N1	GCTGCCCGAGCAATGACTAT
fccF-N2	GACTTGCAGACTTCGGGCAT
azgA-FW	ACTTTCAAAGCCGCGACAAC
fccF-RT-FW	CCGTGGCCAAAATGAACGCGTA
fccF-RT-RV	TCACTAAGGAATGGAGCAGGCCC

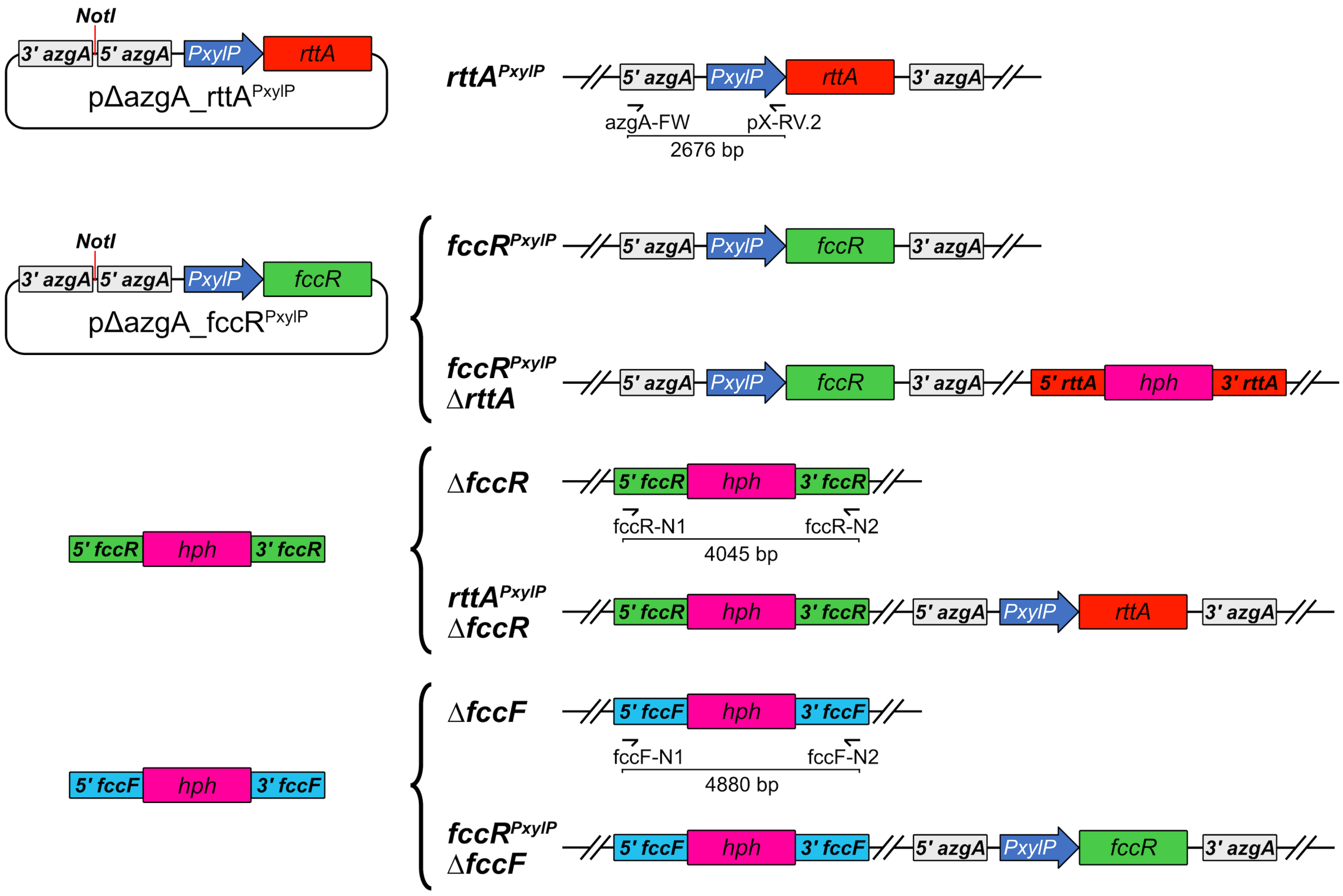

Fig. S1 **Generation of *A. fumigatus* mutants.** The plasmids and the fusion PCR products used to generate the mutants are shown on the left. The corresponding resulting *A. fumigatus* genotypes are shown on the right. Primer pairs used to PCR validate integration of each fragment are shown in the scheme with the resulting length of the product.

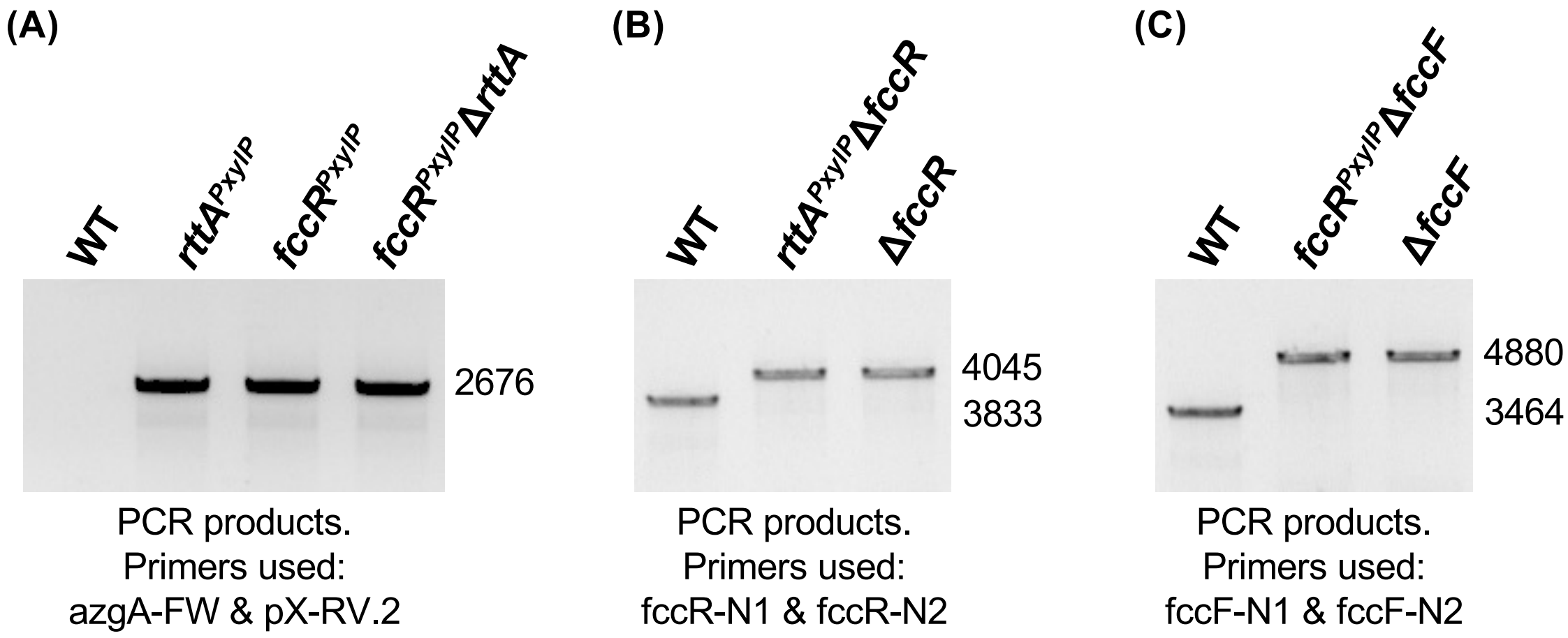

Fig. S2 **PCR validation of overexpression and deletion mutants.** (A) The integration of *rttA* and *fccR* overexpression cassettes into the *A. fumigatus* genome was confirmed by PCR amplification of the 5′ *azgA* region together with the downstream *PxylP*, yielding a 2676 bp product that was detected on an agarose gel. WT served as negative control. (B, C) Deletion of the *fccR* and *fccF* genes in the *A. fumigatus* genome was independently confirmed by PCR amplification of regions spanning the corresponding 5′ and 3′ flanks. In each case, the deletion locus yields a larger PCR product than the WT because the native gene was replaced by longer hygromycin B resistance cassette.
